# Altered transcriptome-proteome coupling indicates aberrant proteostasis in Parkinson’s disease

**DOI:** 10.1016/j.isci.2023.105925

**Published:** 2023-01-04

**Authors:** Fiona Dick, Ole-Bjørn Tysnes, Guido W. Alves, Gonzalo S. Nido, Charalampos Tzoulis

**Affiliations:** 1 Neuro-SysMed Center of Excellence for Clinical Research in Neurological Diseases Department of Neurology, Haukeland University Hospital Department of Clinical Medicine, University of Bergen, 5021 Bergen, Norway; 2 K.G Jebsen Center for Translational Research in Parkinson’s disease, University of Bergen, Bergen, Norway; 3 The Norwegian Center for Movement Disorders and Department of Neurology, Stavanger University Hospital, Stavanger, Norway; 4 Department of Mathematics and Natural Sciences, University of Stavanger, Stavanger, Norway

**Keywords:** Proteomics, Transcriptomics

## Abstract

Aberrant proteostasis is thought to be implicated in Parkinson’s disease (PD), but patient-derived evidence is scant. We hypothesized that impaired proteostasis is reflected as altered transcriptome-proteome correlation in the PD brain. We integrated transcriptomic and proteomic data from prefrontal cortex of PD patients and young and aged controls to assess RNA-protein correlations across samples. The aged brain showed a genome-wide decrease in mRNA-protein correlation. Genes encoding synaptic vesicle proteins showed negative correlations, likely reflecting spatial separation of mRNA and protein into soma and synapses. PD showed a broader transcriptome-proteome decoupling, consistent with a proteome-wide decline in proteostasis. Genes showing negative correlation in PD were enriched for proteasome subunits, indicating accentuated spatial separation of transcript and protein in PD neurons. In addition, PD showed positive correlations for mitochondrial respiratory chain genes, suggesting a tighter regulation in the face of mitochondrial dysfunction. Our results support the hypothesis that aberrant proteasomal function is implicated in PD pathogenesis.

## Highlights

• Proteome-wide decoupling between transcript and protein levels in the aged brain
• Altered pattern of age-dependent decoupling in the Parkinson’s disease (PD) brain
• Increased negative correlation for proteasomal subunits in PD brain
• Negative correlation suggests spatial polarization between protein and its transcript

## Graphical abstract

**Figure:**
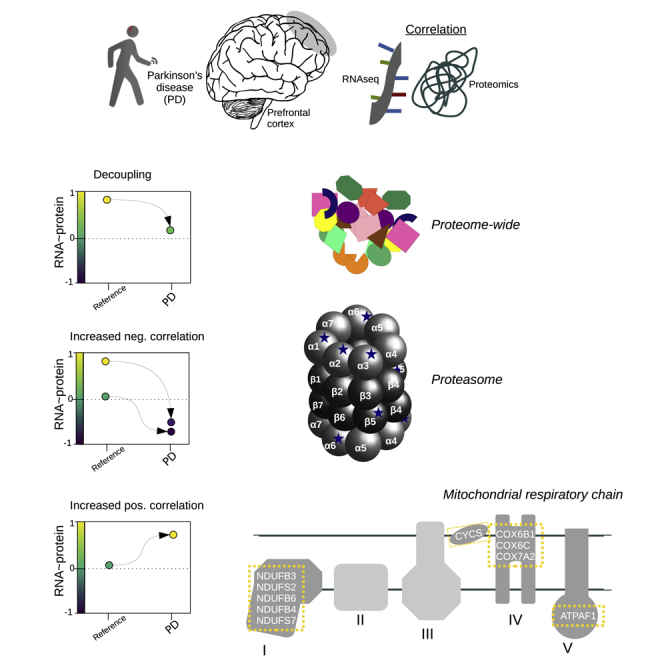

## Introduction

Despite the hierarchical organization of gene expression, the relationship between transcript and protein levels is highly variable in mammalian cells, both across genes and across individuals. Imperfect correlations between mRNA and protein levels are commonly attributed to regulatory mechanisms acting downstream of transcription and influencing the rate of protein synthesis and degradation.^1^^,^^2^^,^^3^ The balanced interplay between these regulatory mechanisms is crucial for maintaining cellular proteostasis.

It was recently shown that the correlation between mRNA and protein levels declines with age in the human brain, possibly because of altered post-transcriptional regulation^4^^,^^5^ and declining proteostasis.^1^ Impaired proteostasis is thought to contribute to the misfolding and aggregation of proteins observed in neurons and other postmitotic cells with ageing,^1^ a phenomenon that is substantially more pronounced in age-dependent neurodegenerative proteinopathies, such as Parkinson’s disease (PD) and Alzheimer’s disease.^6^^,^^7^ In PD, the accumulation of intracellular inclusions containing aggregated forms of the protein alpha-synuclein^8^ has been hypothesized to be due to decreased function of the autophagy-lysosomal pathway.^9^ This is further supported by the fact that mutations in *GBA*, encoding the lysosomal enzyme glucocerebrosidase, greatly increase the risk of PD.^10^ Altered mRNA levels encoding proteasomal components have been consistently found in transcriptomic studies of the PD brain, suggesting that dysfunction of the ubiquitin-proteasome system may also play a role.^11^

We hypothesized that if impaired proteostasis occurs in PD, this should be reflected in the form of altered correlation between the transcriptome and proteome in the patients' brain compared to healthy aged individuals (HA). To test our hypothesis, we performed transcriptome and proteome-wide analyses using RNA sequencing and proteomics in the brain of 15 PD patients and 9 demographically matched healthy controls and assessed the correlation between the levels of each transcript and its cognate protein. Because it is known that extensive changes leading to mRNA-protein decoupling occur with ageing in the human brain,^4^^,^^5^ we also analyzed brain samples of four individuals in early infancy. Ageing remains the strongest known PD risk factor, and this additional group allowed us to compare changes in mRNA-protein correlations arising because of neurologically healthy ageing from those that are specific to pathological ageing with PD.

Our results show that the PD brain is characterized by genome-wide altered mRNA-protein correlation compared to neurologically healthy ageing. The pattern of this altered relationship between transcriptome and proteome is highly consistent with a disease-related impairment in proteostasis.

## Results

### Brain RNA and protein expression patterns are highly distinct between young infants and aged individuals

**Figure 1:**
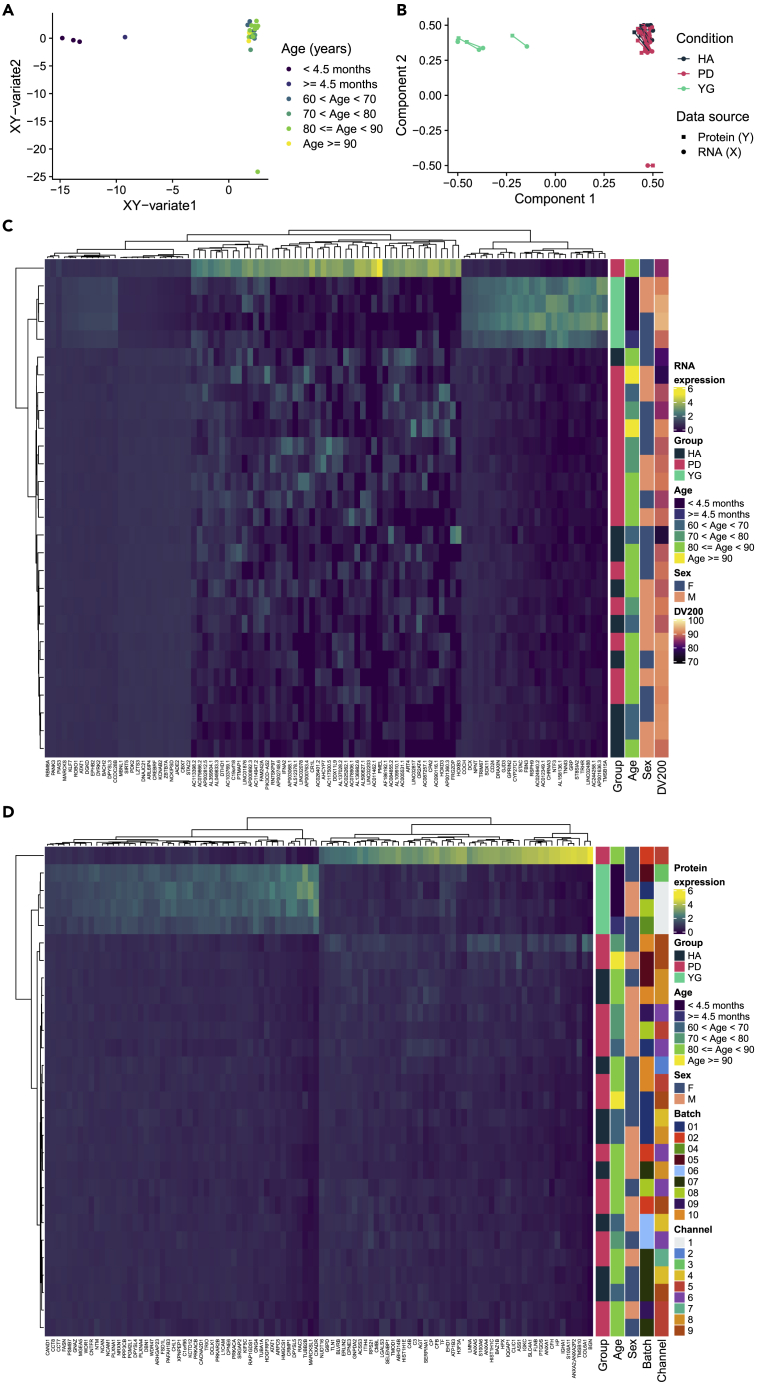
Integrative analysis of age-specific expression patterns in the transcriptome and proteome using sPLS (A) Data points (samples) colored by age in years (binned) in the combined XY variate space defined by the results of sPLS dimensionality reduction analysis (see STAR Methods: “Covariance between omic layers”). Coordinates of samples are the mean over the coordinates in the subspaces of X and Y. (B) Samples plotted separately in the subspaces X (circle) and Y (square) spanned by their first two components. Color coding indicates group membership (HA: dark blue; PD: pink; YG: turquoise); shape indicates omic layer (protein expression: square; transcript expression: circle). (C and D) Heatmap displaying the expression (scaled) of selected features (from sPLS) of components I and II from the transcriptome dataset (C) and the protein dataset (D). Row annotations visualize sample specific metadata.

We mapped the transcriptome and proteome in prefrontal cortex tissue from 4 young infants (YG), 9 neurologically healthy aged individuals (HA), and 15 individuals with idiopathic PD (Table S1). First, we assessed the overall expression pattern of the groups by integrating gene expression (X, N = 29,959 genes) with protein expression (Y, N = 2,953 proteins). Using sparse Partial Least Squares regression (sPLS), we were able to reduce dimensionality for both X and Y and project the samples in an unsupervised manner onto the combined XY-variate space. The YG group was markedly separated from the aged groups HA and PD according to their biological characteristics in the combined variate space (cluster median silhouette width:YG = 0.79; HA = 0.54; PD = -0.49, Euclidean distance; Figure 1A) as well as in the separated variate space (Figure 1B), meaning that the group separation was independent of whether the selected features were restricted to either the transcriptome or the proteome, with both datasets strongly agreeing. The first XY-variate was strongly correlated with age (r = 0.95, p = 3.23 × 10^−14^, Pearson). The expression of the N = 50 selected features for each component (I, II) of X (RNA) and Y (Protein) is visualized in heatmaps in Figures 1C and 1D, respectively, showing a clear clustering of the YG group.

### The transcriptome-proteome correlation signature is altered in the aged brain

**Figure 2:**
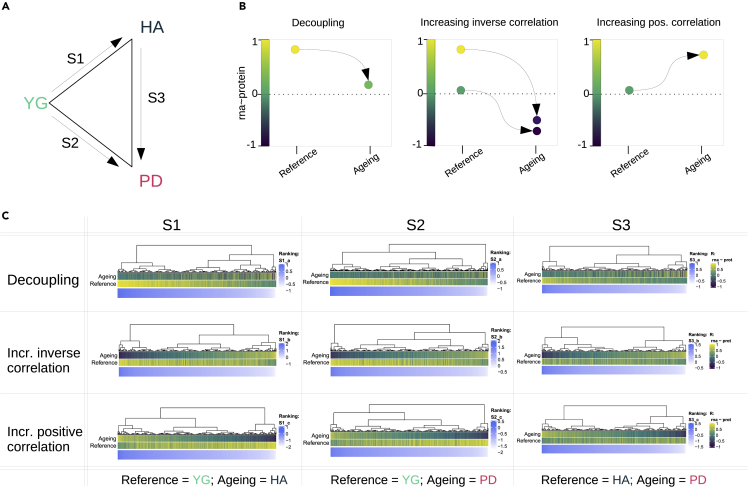
Comparisons and gene score ranking for gene-set enrichment analysis (A) Schematic illustration of comparisons between groups. Each comparison is between a reference group and an ageing group (either HA or PD). For S1, we define YG as the reference and HA as the ageing group. Similarly, for S2, we define YG as a reference and PD as the ageing group. Finally, in S3 we investigate the differences between HA (reference) and PD (ageing). PD: Parkinson's disease; HA: healthy aging; YG: infants. (B) Schematic representation of correlation changes: i) decoupling ii) increasing inverse correlation and iii) increasing positive correlation. We calculated scores to rank genes according to each of these three trends to perform change-specific pathway enrichment analysis. (C) Gene scores calculated for the three comparisons (as defined in A) and correlation trends (as defined in B) displayed in blue, mapped to the respective reference and ageing correlation coefficient. The correlation coefficients are colored from −1 (dark blue) to zero (green) to 1 (yellow).

Because mRNA and protein levels are known to be tightly correlated during neurodevelopment,^4^ we leveraged the YG group as a control outgroup to assess alterations that occur between early development and old age. We assessed differences in correlation coefficient distributions between YG and HA, YG and PD and HA and PD (Figure 2A). We characterized changes in correlation depending on the direction of change: (1) Decoupling: correlation decreases towards zero, (2) increased negative (inverse) correlation and (3) increased positive correlation (Figures 2B and 2C).

To compare the transcriptome-proteome coupling between YG and HA groups, we calculated gene-wise correlation coefficients (r, Pearson) across samples in each of the groups (r_YG_ and r_HA_ for the YG and HA group, respectively) for N = 2,104 genes (Table S2). We will henceforth use the term *gene* for both the gene and the protein it encodes.

**Figure 3:**
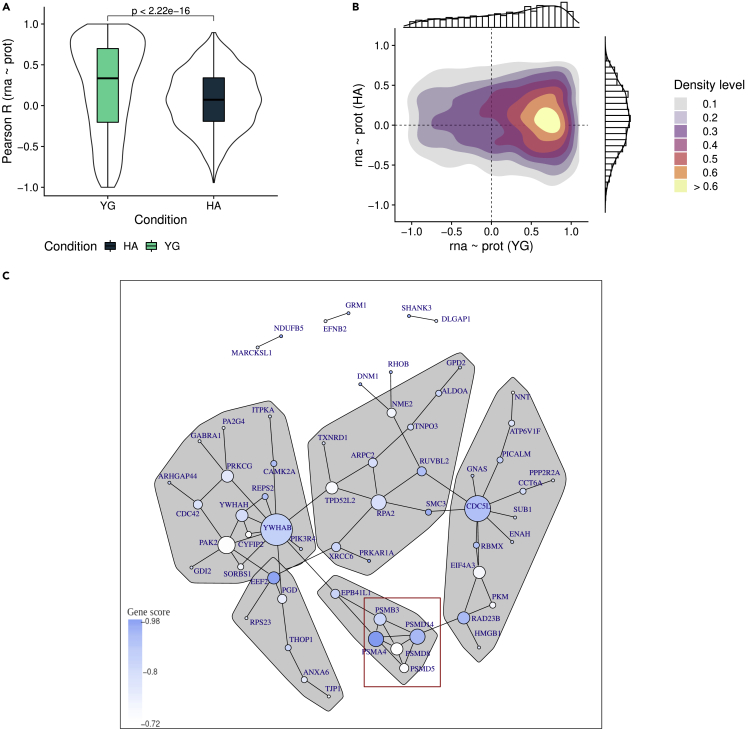
Decoupling of transcriptome and proteome in neurologically healthy aged individuals (A) Distribution of gene-wise correlation coefficients for the groups YG (turquoise) and HA (dark blue) (Wilcoxon unpaired test). (B) Two-dimensional density plot displaying within-gene mRNA-protein Pearson correlations in YG (x-axis) versus HA (y-axis). (C) Protein-protein interaction (PPI) network for genes in the 0.90 quantile of gene-scores (blue), ranking genes by decoupling in HA. Only genes that have at least one edge are displayed. Vertex communities were identified using edge betweenness (R package igraph). Only communities with more than 5 members are displayed. PPI is based on coexpression, experimental evidence of interaction and neighbourhood characteristics.

As expected, transcriptome-proteome correlation in YG was significantly higher compared to that of HA as shown by the transcript-protein r distributions (median r_YG_ = 0.34; median r_HA_ = 0.07; p < 2 × 10^−16^, Wilcoxon) (Figure 3A).

To further characterize the differences in the transcriptome-proteome coupling, we generated a two-dimensional density plot of the gene-wise transcript-protein correlations (Figure 3B). Most genes exhibited a high correlation in YG (r_YG_> 0.5) and a lack of correlation in HA (r_HA_∼0). We henceforth refer to this age-dependent decrease in correlation as *decoupling* (Figure 2B). Additional high-density areas were observed for genes with low absolute correlation in both groups, and for genes transitioning from a highly positive correlation in YG to a negative correlation in HA. Finally, very few genes showed an age-dependent increase in correlation. These observations indicate that most genes show a tight positive correlation between mRNA and protein levels during early infancy. In the HA group, however, this correlation either decreases towards zero (r_HA_∼0, decoupling) or becomes negative/inverse (r_HA_< 0, increased anticorrelation).

### Altered mRNA-protein correlation in the aged brain is enriched for specific biological functions

Next, we assessed whether altered mRNA-protein correlation in the HA group is enriched for specific pre-defined biological pathways. To this end, we divided genes into three groups according to their changes in correlation (YG->HA): a) decoupled (r_YG_> 0, r_HA_∼0), b) increased inverse correlation (r_YG_> 0, r_HA_< 0), c) increased positive correlation (r_YG_> 0, r_HA_> r_YG_). Genes in each group (Figure 2B) were ranked according to the magnitude of the difference (δ (r_HA_,r_YG_)) (Figure 2C). Although most genes showed decoupling in HA, we found no significant enrichment in this group for any specific biological pathway. A protein-protein interaction network of the top decoupled genes (score >90% quantile, N = 61) with at least one edge to another gene, revealed 5 interconnected groups with more than 5 members (Figure 3C), strongly suggesting a functional relationship. Notably, 5 of the 6 members of one of these groups were subunits of the proteasome complex (*PSMA4*, *PSMB3*, *PSMD5*, *PSMD8*, and *PSMD14*). The gene group with increased inverse correlation showed significant enrichment for the GO geneset “synaptic vesicle” (adjusted p = 0.002). Finally, genes which showed increased positive correlation from YG to HA showed no significant enrichment.

### The age-dependent decoupling between mRNA and protein levels is more pronounced in the PD brain

**Figure 4:**
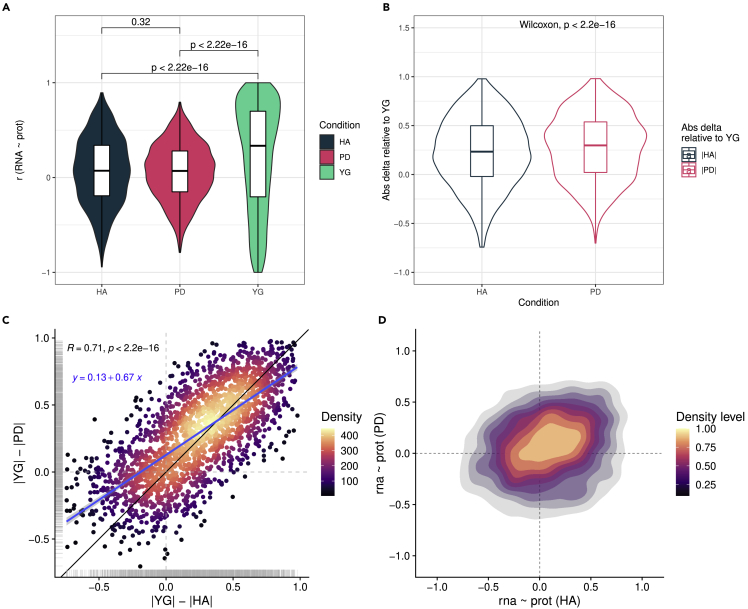
Altered correlation coefficient distribution in PD (A) mRNA-protein correlation distributions for HA (darkblue), PD (pink) and YG (turquoise) (Wilcoxon unpaired test). (B) Distribution of the deltas (differences in absolute correlation coefficients) between the reference (YG) and HA (dark blue), and YG and PD (pink) (Wilcoxon paired test). (C) Relationship between δ_age_ = |r_YG_| - |r_HA_| (x-axis) and δ_PD_ = |r_YG_| - |r_PD_| (y-axis). Color indicates data point density. Blue line indicated the linear model fit (y ∼ x). Black line is the diagonal (intercept = 0, slope = 1). (D) Two-dimensional density plot displaying both distribution and relationship between the RNA ∼ prot protein correlations in HA (x-axis) and PD (y-axis).

Next, we wanted to assess how the transcriptome-proteome coupling changes in PD compared to that in normal, neurologically healthy aged individuals. Correlation distributions for PD and HA groups showed no significant difference (p = 0.52, Wilcoxon) with a median close to zero for both groups (median r_PD_ = 0.070, median r_HA_ = 0.072). However, PD showed an overall lower variance (σ^2^ (r_HA_) = 0.13, σ^2^ (r_PD_) = 0.09) and a reduced range (range r_HA_ = [-0.94, 0.89]; range r_PD_ = [-0.79, 0.79]), indicating a more pronounced trend of decoupling (Figure 4A).

To further investigate this, we calculated the absolute difference in the gene-wise transcript-protein correlation between YG and either HA (δ_age_ = |r_YG_|-|r_HA_) or PD (δ_PD_ = |r_YG_|-|r_PD_|). Of interest, the two distributions differed significantly (p = 2.2 × 10^−16^, Wilcoxon, paired, Figure 4B), with δ_PD_ (median = 0.30) being higher than δ_HA_ (median = 0.23). These findings indicate that the age-dependent loss of transcript-protein correlation is likely more pronounced in pathological aging with PD than in the HA group, as evident also by the r_HA_∼r_YG_ and r_PD_∼r_YG_ density distributions (Figure S3A). Despite these differences, δ_age_ and δ_PD_ showed a highly significant positive correlation (r = 0.71, p < 2.2 × 10^−16^, Pearson) (Figure 4C), suggesting that the process of decoupling is qualitatively similar and has a comparable genome-wide distribution in HA and PD, although it is more pronounced in the latter.

### Altered transcript-protein correlation in the PD brain is enriched for specific biological processes

Like HA, the PD group showed a general trend for decoupling compared to YG (Figure S3A) and no significant enrichment for specific pathways. The protein-protein interaction network of top decoupled genes (N = 53 nodes), revealed 5 interconnected groups with more than 5 members (Figure S3B), strongly suggesting a functional relationship. Three of these groups consist of genes related to synapse components and function. One group includes 4 proteasomal subunits, similar to the results in HA.

Genes showing increased inverse correlation with PD ageing were significantly enriched for “KEGG Proteasome” and 28 GO pathways mainly related to protein degradation including proteasome complex, ubiquitination and unfolded protein response (FDR <0.05, Table 1). Genes showing increased positive correlation with PD ageing were not significantly enriched for any genesets.

**Table 1:** Pathway enrichment results on differences in correlation coefficients between groups

Comparison	Score-type	Pathway	Adj. p-value	Enrichment score	Permutation p-value
YG- > HA	Incr. neg. correlation	synaptic vesicle	0.00172	0.45225	0.0830
YG- > PD	Incr. neg. correlation	catalytic activity acting on DNA	0.02543	0.63176	0.0018
YG- > PD	Incr. neg. correlation	lyase activity	0.03840	0.42692	0.0030
YG- > PD	Incr. neg. correlation	double-stranded RNA binding	0.03065	0.57149	0.0074
YG- > PD	Incr. neg. correlation	Proteasomal ubiquitin-independent protein catabolic process	0.00475	0.67097	0.0100
YG- > PD	Incr. neg. correlation	cellular response to toxic substance	0.04809	0.43823	0.0138
YG- > PD	Incr. neg. correlation	negative regulation of canonical Wnt signaling pathway	0.00475	0.51380	0.0168
YG- > PD	Incr. neg. correlation	ATPase activator activity	0.04809	0.60046	0.0178
YG- > PD	Incr. neg. correlation	translation initiation factor activity	0.01163	0.57836	0.0196
YG- > PD	Incr. neg. correlation	positive regulation of canonical Wnt signaling pathway	0.00443	0.53483	0.0204
YG- > PD	Incr. neg. correlation	regulation of hematopoietic progenitor cell differentiation	0.00443	0.56518	0.0274
YG- > PD	Incr. neg. correlation	peptidase complex	0.00475	0.49484	0.0276
YG- > PD	Incr. neg. correlation	anaphase-promoting complex-dependent catabolic process	0.00443	0.56525	0.0348
YG- > PD	Incr. neg. correlation	interleukin-1-mediated signaling pathway	0.00689	0.50992	0.0376
YG- > PD	Incr. neg. correlation	tumor necrosis factor-mediated signaling pathway	0.00475	0.52302	0.0408
YG- > PD	Incr. neg. correlation	response to interleukin-1	0.01099	0.45318	0.0430
YG- > PD	Incr. neg. correlation	innate immune response-activating signal transduction	0.01006	0.44820	0.0438
YG- > PD	Incr. neg. correlation	NIK/NF-kappaB signaling	0.00475	0.54559	0.0492
YG- > PD	Incr. neg. correlation	innate immune response activating cell surface receptor signaling pathway	0.00689	0.50023	0.0496
YG- > PD	Incr. neg. correlation	non-canonical Wnt signaling pathway	0.00565	0.48994	0.0500
YG- > PD	Incr. neg. correlation	antigen processing and presentation of peptide antigen via MHC class I	0.00443	0.54314	0.0528
YG- > PD	Incr. neg. correlation	regulation of RNA stability	0.00946	0.45440	0.0568
YG- > PD	Incr. neg. correlation	negative regulation of cell cycle G2/M phase transition	0.00683	0.52755	0.0644
YG- > PD	Incr. neg. correlation	regulation of cellular amine metabolic process	0.01041	0.46303	0.0650
YG- > PD	Incr. neg. correlation	regulation of DNA-templated transcription in response to stress	0.00689	0.48683	0.0722
YG- > PD	Incr. neg. correlation	regulation of morphogenesis of an epithelium	0.02583	0.45410	0.0772
YG- > PD	Incr. neg. correlation	SCF-dependent proteasomal ubiquitin-dependent protein catabolic process	0.01652	0.48316	0.0956
YG- > PD	Incr. neg. correlation	antigen processing and presentation of peptide antigen	0.03672	0.38724	0.1340
YG- > PD	Incr. neg. correlation	positive regulation of ATPase activity	0.04809	0.46536	0.1408
YG- > PD	Incr. neg. correlation	KEGG_PROTEASOME	0.00376	0.57805	0.0336
HA- > PD	Incr. neg. correlation	KEGG_PROTEASOME	0.02790	0.61400	0.0210

Pathways are sorted within each ranking comparison by their enrichment score. Permutation p-value represents the fraction of permutations (from randomly sampled data) for which the enrichment score was greater than the enrichment score of the observation.

Comparing YG and PD cannot confidently differentiate ageing- from disease-related changes, hence we also performed a direct comparison between HA and PD. These analyses revealed an altered profile of transcript-protein correlation in PD compared to HA (Figure 4D). Genes showing increased decoupling in PD and genes showing increased positive correlation in PD were not significantly enriched for any genesets. Genes showing increased inverse correlation in PD were enriched for “KEGG Proteasome” (FDR <0.05, Table 1). Similar to the comparison with the YG group, this enrichment was driven primarily by proteasomal subunits. The magnitude of anticorrelation varied substantially, affecting certain proteasomal subunits more than others (median(r) = −0.32, range(r) = [-0.68,0.29], σ^2^(r) = 0.07, N = 29).

### Negatively and positively correlated genes in PD are enriched for proteasome complex and cellular respiration respectively

To differentiate changes in healthy aged individuals from those occurring in ageing with PD, we ranked genes according to the difference in correlation between groups. In addition, we were interested to see whether the most positively and the most negatively correlated genes (max(r) and min(r)) within a group (irrespective of the difference between groups) were enriched for specific pathways. Pathway analysis on gene lists ranked by both positive (r) and negative (–r) correlation in YG showed no enrichment. HA, showed no significantly enriched pathway for positively correlated genes. Negatively correlated genes in HA were enriched for pathways related to the ribosome and synaptic vesicle (Table S3), the latter being in line with the results of the YG->HA comparison, where we found this enrichment for genes that are increasingly negatively correlated in HA vs YG (Table 1). Finally, in PD, positively correlated genes were enriched for cellular respiration and neurodegenerative diseases, including Parkinson’s disease (Table S3). The enrichment for both of these is driven by genes of the mitochondrial respiratory chain (MRC). Negatively correlated genes in PD were enriched for the proteasome and related pathways (Table S3), in line with the results of our main analysis, where we found this enrichment for genes that show increased negative correlation in PD in comparison to both YG and HA (Table 1).

**Figure 5:**
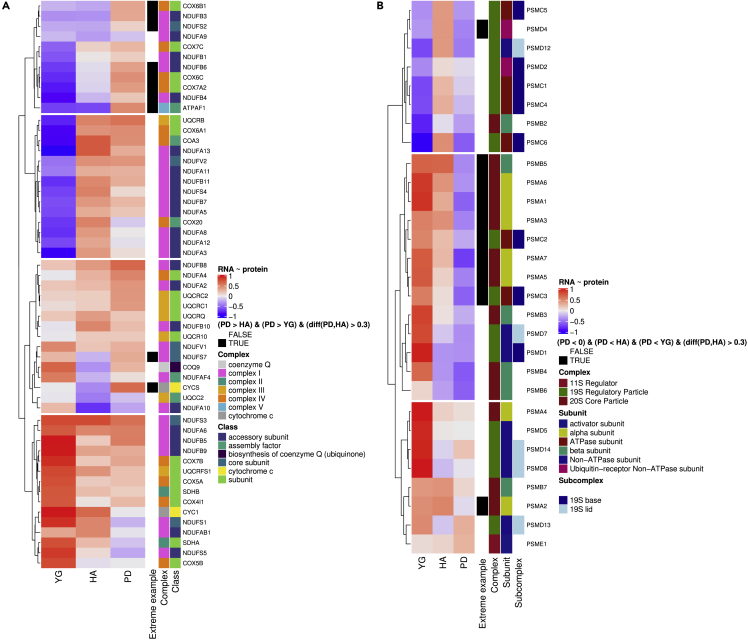
Diverse correlation patterns across subunits of proteasome complex and complexes of the MRC (A) RNA ∼ protein correlation coefficients for genes encoding proteins of the MRC (rows) for the groups YG, HA and PD (columns). Color coding of the heatmap shows negative correlation in blue, no correlation in white and positive correlation in red. Color saturation reflects magnitude of the correlation coefficient. Row annotations visualize gene-specific information: “Complex” describes which complex of the MRC the gene belongs to; “Class” describes what kind of subunit or factor the gene encodes; genes for which r_PD_ is greater than r_HA_ and r_YG_ and the difference between r_PD_ and r_HA_ is greater than 0.3 are marked as extreme examples (black) of positive correlation in PD. (B) RNA ∼ protein correlation coefficients for genes encoding proteins of the proteasome complex (rows) for the groups YG, HA and PD (columns). Color coding of the heatmap shows negative correlation in blue, no correlation in white and positive correlation in red. Color saturation reflects magnitude of the correlation coefficient. Row annotations visualize gene-specific information: “Complex” describes which complex of the proteasome the gene belongs to; “Subunit” classifies the gene based on the type of subunit it encodes; “Subcomplex” indicates whether genes of the 19S regulatory particle belong to the base or lid subcomplex; genes for which r_PD_ is negative, less than r_HA_ and r_YG_ and the difference between r_PD_ and r_HA_ is greater than 0.3 are marked as extreme examples (black) of negative correlation in PD.

Based on this, we investigated the correlation pattern of individual genes of the proteasome complex and the MRC. For both we found a wide range of correlations, and genes driving the enrichment were not specific to one type of proteasomal subunit or one type of MRC complex (Figure 5). For example, genes encoding MRC proteins with increased positive correlation in PD do not show a specific pattern related to complexes but span complex I, IV and V (Figure 5A). Similarly, genes that are negatively correlated in PD but not- or less in YG and HA encode proteins of both the 20S core and 19R regulatory particle of the proteasome, including alpha and beta subunits (Figure 5B).

### Limitations due to unbalanced group sizes

This study is limited by unbalanced group sizes (N_YG_ = 4, N_HA_ = 9, N_PD_ = 15), possibly affecting the statistics of the analyses. To investigate this, we created permutations and analyzed correlation patterns on these. Specifically, for each permutation we sampled individuals with replacement from the complete population (YG, HA, PD) and formed groups with the same sizes as the original groups (k = 4, k = 9 and k = 15 samples, respectively). We found both mean range and variance of correlation coefficients overall permutations were smallest in the k = 15 group (median_r_k=4_ = 0.27, median_r_k=9_ = 0.28, median_r_k=15_ = 0.26; σ^2^(r_k=4_) = 0.06, σ^2^(r_k=9_) = 0.02, σ^2^(r_k=15_) = 0.01; range(r_YG_) = [-1,1], range(r_HA_) = [-0.12, 0.61], range(r_PD_) = [-0.03, 0.52]; N permutations = 5000, Figure S4A). Furthermore, for each permutation, we calculated the absolute difference in gene-wise transcript-protein correlation between two groups δ_k=9_ = |r_k=4_|-|r_k=9_|, δ_k=15_ = |r_k=4_|-|r_k=15_|), reflecting the group sizes of our original analysis (δ_age_ = |r_YG_|-|r_HA_|), (δ_PD_ = |r_YG_|-|r_PD_|), and investigated the effect of group size on the delta. The mean delta overall genes and permutations for k = 15 was greater (δ_k=15_ = 0.21) than that of k = 9 (δ_k=9_ = 0.16), suggesting that both variance of correlation and difference in absolute correlation (δ) between two groups is likely influenced by the group size, where the biggest group (PD, k = 15) shows the smallest variance and the greatest delta when compared to a group of smaller size (YG, k = 4). To investigate the effect of this on pathway analysis, we calculated gene rankings on the permuted dataset for the three functional scenarios: a) decoupling, b) increasing inverse correlation and c) increasing positive correlation. In each permutation we compared two groups resembling our original comparisons, i.e.: 1) YG->HA: k = 4->k = 9; 2) YG->PD: k = 4->k = 15; and 3) HA->PD: k = 9->k = 15. The enrichment scores of 29 out of 31 originally significant pathways (Table 1) were greater than 90% of the permutation enrichment scores (N permutations = 5,000). Both of our two main findings: (1) genes that increase in negative correlation in HA compared to YG are enriched for “synaptic vesicle” and (2) genes that increase in negative correlation in PD compared to both YG and HA are enriched for genes of the proteasome complex, were significant in the permutation test (permutation p-value <0.1, Table 1). These findings are thus more likely to be associated with the condition of the groups (YG, HA, PD), rather than the result of unbalanced sample sizes.

## Discussion

Here, we assess for the first time the genome-wide transcriptome-protein correlation in the PD brain, compared to neurologically healthy age-matched individuals. In line with previous reports in yeast,^12^ fish,^13^ macaque and humans,^4^^,^^5^ the correlation was significantly lower in the aged individuals compared to the infants, consistent with an age-dependent decoupling between transcript and protein abundance. Previous studies have suggested that this age-dependent decoupling in the brain may preferentially affect certain biological processes, including transcriptional, translational and posttranslational regulation, signaling pathways, and mitochondrial function.^5^^,^^12^^,^^13^ In our data, however, genes that decoupled in the aged group did not exhibit a significant enrichment in any specific biological pathways, suggesting that the age-dependent loss of correlation between mRNA and protein is a general, genome-wide process not related to particular functions.

The phenomenon of age-dependent decoupling between mRNA and protein suggests that in the infant brain protein abundance is determined mainly by transcript concentration, whereas in the aged brain, modulating the rates of translation and protein degradation assumes a more central role in determining protein abundance than transcriptional regulation. At the same time, the tight correlation observed between mRNA and protein levels in the neonatal brain may be, at least partly, also related to the ongoing proliferation and migration of glial progenitors,^14^ a process heavily dependent on transcriptional regulation via the binding of a broad spectrum of transcription factors.^15^

In addition to the physiological effects of brain development, the mRNA-protein decoupling observed in the aged brain may reflect pathological changes taking place in ageing post-mitotic cells. A decline in proteasome function with ageing has been shown in multiple mammalian tissues and is believed to be contributing to the accumulation of misfolded and damaged proteins in the ageing brain (reviewed in^16^). Notably, several subunits of the proteasomal complex were among the top decoupled genes. These findings provide further support to the hypothesis of declining and/or aberrant proteasomal function in the aged brain.

Although substantial decoupling was seen in both the HA and PD groups, the distribution of RNA-protein correlation in PD showed an overall lower variance and range, indicating a more pronounced trend of decoupling, compared to HA. Although our data cannot elucidate the molecular mechanisms underlying this phenomenon, a state of heightened decoupling is consistent with disease-related impairment in proteostasis because of altered proteasomal and/or lysosomal function, both of which have been implicated in the pathogenesis of PD by numerous studies.^17^^,^^18^^,^^19^ Thus, our findings support the hypothesis that aberrant proteostasis contributes to the pathogenesis of PD.

In the healthy aged brain, we identified a group of genes exhibiting inverse correlation between transcript and protein levels. This can be potentially explained by the highly polarized cellular architecture of neurons, which allows spatial separation between mRNA and protein.^20^ Although some proteins are translated locally at their resident site, others are synthesized in the soma and transported along the axon/dendrites to their target location. This leads to a steady state in which the transcript resides in the soma, whereas most of the protein is either under transport in the axon or at the synapsis.^20^ Because brain tissue samples typically vary in relative grey/white matter content and therefore also in relative soma/axonal content,^21^ readouts of transcript and protein levels for these genes will be anticorrelated across samples. Specifically, samples enriched in somas will indicate a high relative transcript/protein ratio, whereas samples enriched in axons will indicate a low relative transcript/protein ratio. In line with this hypothesis, genes showing negative correlation in HA were significantly enriched for synaptic vesicle related pathways. These were indeed shown to be preferentially translated in the cell body and then undergo axonal transport to the synapses,^22^^,^^23^ consistent with a spatial compartmentalization of transcripts and their protein products. The top negatively correlated genes in HA were highly positively correlated in the infants, which may reflect a more homogenous distribution of somata and axons and/or reduced axonal transport during development, likely because of immature neuronal morphology.^24^^,^^25^

Of interest, genes showing inverse mRNA-protein correlation in PD were not significantly enriched in synaptic function compared to HA. At least two factors may contribute to this phenomenon. First, disruption of axonal transport has been shown to occur in the PD brain (see^26^ for a review). This would decrease the spatial separation between transcript and protein, thereby blunting the negative correlation across samples. Second, the PD brain, including the prefrontal cortex, is characterized by neuronal and synaptic loss and a relative increase in glial populations.^21^ It is therefore conceivable that if the anticorrelation signal originates from neurons, it may be diluted because of these changes in cellular composition.

Genes showing inverse mRNA-protein correlation in PD were enriched for subunits of the proteasomal complex compared to both infants and neurologically healthy aged individuals. This suggests that these proteins become specifically more polarized in PD, with an accentuated spatial separation of transcript and protein between soma and axon. The ubiquitin-proteasome system has a crucial role in maintaining synaptic proteostasis and modulating neurotransmission and has been shown to be enriched at the synapses.^27^^,^^28^^,^^29^^,^^30^ Moreover, studies in mice have shown that some proteasomal subunits are translated locally at the synapses, whereas others are translated in the soma and transported to the synapses.^23^^,^^31^ Our data indicate that the spatial mRNA-protein separation is uneven across the proteasomal subunits, suggesting a potentially altered stoichiometry of the synaptic proteasome in PD neurons. Loss of, or alternations in stoichiometry of protein complexes with ageing have been reported in killifish,^13^ where the authors suggested that this contributes to impaired proteostasis. Furthermore, the formation of an alternative proteasome complex consisting of an additional alpha-4 subunit (*PSMA7*) in place of an alpha-3 (*PSMA4*) has been shown to be involved in cellular adaptation to environmental stress.^32^ These subunits showed a marked disparity in their correlation values in the PD brain (r_PSMA7_ = −0.68; r_PSMA4_ = 0.06).

Finally, although genes with increased transcript-protein correlation in HA or PD compared to YG showed no functional enrichment, the top-ranked positively correlated genes in PD were significantly enriched for subunits of the MRC, which was not observed in HA. Moreover, the PD brain exhibited an uneven correlation pattern for MRC genes, similar to the disparity we observed among subunits of the proteasome complex and in line with reported age-specific stoichiometric imbalances of protein complexes.^13^

We hypothesize that this positive correlation reflects a decrease in spatial separation of MRC transcripts and proteins in the PD brain. Neurons depend on constant mitochondrial dynamics and motility to ensure that functional mitochondrial populations reside at regions of high energy demand such as synapses.^33^ It has been suggested that neuronal mitochondrial motility and axonal transport decline with aging and neurodegeneration.^34^ As a result, damaged synaptic mitochondria may be less amenable to retrograde transport and, therefore, depend on on-site repair via local translation of nuclear encoded mitochondrial transcripts.^35^ Multiple nuclear-encoded mitochondrial mRNAs have indeed been found to be enriched at synapses,^36^ including mRNA of cytochrome c oxidase subunit 4. In our data we found high positive correlation for genes encoding proteins of cytochrome c oxidase (*COX6A1* r_PD_ = 0.45, *COX6C r*_*PD*_*=0*.*43*) and particularly high positive correlation for cytochrome c (*CYCS*) in PD (r_PD_ = 0.62) compared to HA (r_HA_ = −0.35) or YG (r_YG_ = −0.01), suggesting a decreased polarization of transcript and protein for this gene in PD (Figure 5A). Mitochondrial dysfunction is a key feature associated with PD.^37^ Based on these findings, we propose the hypothesis that PD neurons may be more dependent on local translation of specific MRC subunits because of impaired mitochondrial motility and/or dynamics.

In summary, we demonstrate that the PD brain is characterized by altered coupling between the transcriptome and proteome compared to neurologically healthy aged individuals. This altered relationship is consistent with an extensive, possibly proteome-wide, impairment of proteostasis, and supports the hypothesis that aberrant proteasomal function is implicated in the pathogenesis of PD. Moreover, these findings have important implications for the correct interpretation of transcriptomic studies in this field. Gene expression studies are extensively used to identify disease-related pathways in ageing and neurodegeneration, and it is assumed that observed differences in mRNA levels reflect differences at the protein level. If the relationship between transcript and protein is altered in PD, this should be accounted for when interpreting the molecular impact of differential gene expression in the patient brain.

### Limitations of the study

Our findings should be interpreted considering certain limitations. Post-mortem RNA degradation in our samples may partly contribute to low correlations between mRNA and protein. Proteins are generally more resilient to post-mortem degradation and survive for longer periods than RNA. In addition, suboptimal integration of RNA and protein could be because of non-identical samples for RNA sequencing and proteomics. However, samples were derived from the exact same area (immediately adjacent to each other) and were treated identically, thus minimizing the discrepancy as much as possible. Because there is no reason to assume that RNA degradation or sampling bias would be systematically different between our groups, this factor is unlikely to confound our results of differential transcript-protein correlation between groups.

Owing to the lower sensitivity of proteomics, our dataset was constrained to only ∼2,000 proteins and thus, our findings are not necessarily representative of the entire genome.

The sample size for the YG group (N = 4) was small because of the restricted availability of this type of tissue, limiting the generalizability of the ageing-associated findings. Nevertheless, the infant group did recapitulate the previously observed high positive correlation for most genes,^5^ suggesting the samples are representative for transcript-protein correlation in the infant brain. It is possible that that the small range and variance of correlation coefficients in the PD group may be partly because of the unbalanced group sizes. However, using permutations tests, we showed that the pathway enrichment results are unlikely to be influenced by this and are, therefore, likely to be associated with the condition of the group (YG, HA, PD). It should also be noted that although we refer to differences between YG and HA or PD as “age-dependent”, this does not imply that these results are due to the process of ageing per se. The YG group is in a state of early neurodevelopment, which is likely different from that of young adults. Finally, because all PD individuals were using some form of dopaminergic therapy during the last year of life, a drug confounding effect cannot be excluded in our data. It is, however, unlikely that an intervention as specific as dopaminergic stimulation would have such a genome-wide effect in RNA-protein correlation, especially given the fact that there are very few dopaminergic cells in the PFC.

## STAR★Methods

### Key resources table

**Table:** 

REAGENT or RESOURCE	SOURCE	IDENTIFIER
**Deposited data**		

RNA sequencing counts, protein intensities and metadata in R file format	This paper	https://doi.org/10.6084/m9.figshare.21617886.v1

**Software and algorithms**		

MaxQuant	Cox et al.^38^	https://www.maxquant.org/
Trimmomatic	Bolger et al.^39^	http://www.usadellab.org/cms/?page=trimmomatic
fastQC	Babraham Institute	http://www.bioinformatics.babraham.ac.uk/projects/fastqc
Salmon	Patro et al.^40^	https://github.com/COMBINE-lab/salmon
R package tximport	Soneson et al.^41^	https://bioconductor.org/packages/release/bioc/html/tximport.html
R package mixOmics,	Rohart et al.^42^^,^^43^	http://mixomics.org/
R package ComplexHeatmaps	Eils et al.^44^	https://www.bioconductor.org/packages/release/bioc/html/ComplexHeatmap.html
R package fgsea	Korotkevich et al.^45^	https://github.com/ctlab/fgsea
R package coexnet	Henao^46^	https://www.bioconductor.org/packages/release/bioc/html/coexnet.html
Database string	Szklarczyk et al.^47^	https://string-db.org/
R package igraph	Csardi^48^	https://igraph.org/r/

**Other**		

Analyses and resources	This paper	https://github.com/fifdick/alt_rna_prot_coupling_PD; https://doi.org/10.5281/zenodo.7357345

### Resource availability

#### Lead contact

Further information and requests for resources should be directed to and will be fulfilled by the lead contact, Charalampos Tzoulis (Charalampos.Tzoulis@uib.no).

#### Materials availability

This study did not generate new unique reagents.

### Experimental model and subject details

#### Cohorts

All experiments were conducted in fresh-frozen prefrontal cortex (Brodmann area 9) tissue from a total of 28 individuals comprising young infants (YG, N = 4, age 0–0.38 years), neurologically healthy aged individuals (HA, N = 9, age 63–88 years) and individuals with idiopathic Parkinson's disease (PD, N = 15, age 69–95 years) from the Park-West study, a prospective population-based cohort which has been described in detail.^49^ Whole-exome sequencing had been performed on all PD patients and known causes of Mendelian PD and other monogenic neurological disorders had been excluded.^50^ Controls had no known neurological disease and were matched for age and sex. Individuals with PD fulfilled the National Institute of Neurological Disorders and Stroke^51^ and the UK Parkinson's disease Society Brain Bank^52^ diagnostic criteria. All PD cases showed neuropathological changes consistent with PD, whereas controls had no pathological evidence of neurodegeneration. Cohort demographics including sex and age of all individuals are listed in Table S1.

Ethical permission for these studies was obtained from our regional ethics committee (REK 2017/2082, 2010/1700, 131.04). Written formal informed consent was obtained from all participants or their next of kin. We confirm that we have read the Journal’s position on issues involved in ethical publication and affirm that this work is consistent with those guidelines.

### Method details

#### RNA sequencing

Total RNA was extracted from prefrontal cortex tissue homogenate for all samples using RNeasy plus mini kit (Qiagen) with on-column DNase treatment according to the manufacturer’s protocol. The final elution was made in 65 μL of dH2O. The concentration and integrity of the total RNA were estimated by Ribogreen assay (Thermo Fisher Scientific), and Fragment Analyzer (Advanced Analytical), respectively and 500ng of total RNA was used for downstream RNA sequencing applications. First, nuclear and mitochondrial rRNA was removed using Ribo-ZeroTM Gold (Epidemiology) kit (Illumina, San Diego, CA) using the manufacturer’s recommended protocol. Immediately after rRNA removal, RNA was fragmented and primed for the first strand synthesis using the NEBNext First Strand synthesis module (New England BioLabs Inc., Ipswich, MA). Directional second strand synthesis was performed using NEBNExt UltraDirectional second strand synthesis kit. Following this, the samples were taken into standard library preparation protocol using NEBNext DNA Library Prep Master Mix Set for Illumina with slight modifications. Briefly, end-repair was done followed by poly(A) addition and custom adapter ligation. Post-ligated materials were individually barcoded with unique in-house Genomic Services Lab (GSL) primers and amplified through 12 cycles of PCR. Library quantity was assessed by Picogreen Assay (Thermo Fisher Scientific), and the library quality was estimated by utilizing a DNA High Sense chip on a Caliper Gx (Perkin Elmer). Accurate quantification of the final libraries for sequencing applications was determined using the qPCR-based KAPA Biosystems Library Quantification kit (Kapa Biosystems, Inc.). Each library was diluted to a final concentration of 12.5nM and pooled equimolar prior to clustering. One hundred twenty-five bp Paired-End (PE) sequencing was performed on an Illumina HiSeq2500 sequencer (Illumina, Inc.). RNA quality, measured by the DV200 score, varied across samples (median YG = 92, median HA = 88, median PD = 89), although the difference between groups was not statistically significant (YG, HA: p = 0.10, HA, PD: p = 1.00, YG, PD: p = 0.10, Wilcoxon rank sum test).

#### Lysis and protein digestion

Tissue samples for proteomics were taken simultaneously from a region immediately adjacent (within 1–5 mm) to the sample used for RNA sequencing. The samples for RNA and proteomics were dissected and stored in the same way and underwent no freeze-thaw cycles. 10 μL of lysis buffer (4% SDS, 0.01 M TRIS pH 7.6) was added to 1mg of brain tissue. The tissue was mechanically lysed using Precellys CK 14 ceramic beads, together with the Precellys Evolution (Bertin Corp, Rockville MD, USA). Lysed tissue was transferred to Eppendorf tubes and heated to 95 ºC for 5 minutes, before centrifugation at 10,000g for 5 minutes. The clarified supernatant was transferred to new Eppendorf tubes. Protein measurement was performed using the Pierce BCA protein assay kit (Thermo Fisher). The samples were mixed with up to 50 μL of the clarified lysate with 200μL of 8M urea in 0.1 M Tris/HCl pH 8.5 in the filter unit (Microcon YM-30 (Millipore, Cat. MRCF0R030)) and centrifuged at 14,000 × g for 30 min and repeated twice. In total 30μg of protein per sample was used. The samples were reduced with 10m M DTT (1h, RT) and alkylated using 50 mM IAA (1h, RT), and digested overnight at 37ºC with 1:50 enzyme: substrate ratio of sequencing grade trypsin (Promega, Madison, WI). Following digestion, samples were acidified with formic acid and desalted using HLB Oasis SPE cartridges (Waters, Milford, MA). Samples were eluted with 80% acetonitrile in 0.1% formic acid and lyophilized. Peptides were stored at −80ºC until use.

#### TMT labeling and fractionation

Digested peptides from each sample were chemically labelled with TMT reagents 10 plex (Thermo Fisher). Peptides were resuspended in a 30 μL resuspension buffer containing 0.1 M TEAB (Triethylammonium bicarbonate). TMT reagents (0.1mg) were dissolved in 41 μL of anhydrous ACN of which 20 μL was added to the peptides. Following incubation at RT for 1 h, the reaction was quenched using 5% hydroxylamine in HEPES buffer for 15minat RT. The TMT-labeled samples were pooled at equal protein ratios followed by vacuum centrifuge to near dryness and desalting using Oasis PRIME HLB cartridges. Peptides were fractionated into 8 fractions using the Pierce High pH Reverse-phase Peptide fractionation kit (Thermo Fisher Scientific). The TMT experiment batch setup included additional samples which were not considered in the analysis but included in the preprocessing (filtering and normalization) of the proteomics data.

#### Liquid chromatography and mass spectrometry analysis

Each sample was freeze-dried in a Centrivap Concentrator (Labconco) and dissolved in 2% ACN, 1% FA. Approximately 0.5 μg of peptides from each fraction was injected into an Ultimate 3000 RSLC system (Thermo Scientific) connected to a Q-Exactive HF equipped with an EASY-spray ion source (Thermo Scientific). The samples were loaded and desalted on a precolumn (Acclaim PepMap 100, 2 cm ∗ 75 μm i.d. nanoViper column, packed with 3 μm C18 beads) at a flow rate of 3 μL/min for 5 min with 0.1% TFA. The peptides were separated during a biphasic ACN gradient from two nanoflow UPLC pumps (flow rate of 0.200 μL/min) on a 50 cm analytical column (PepMap RSLC, 50 cm ∗ 75 μm i.d. EASY-spray column, packed with 2 μm C18 beads (Thermo Scientific). Solvent A was 0.1% FA in water, and Solvent B was 100% ACN. The mass spectrometer was operated in data-dependent acquisition mode to automatically switch between full scan MS1 and MS2 acquisition. The instrument was controlled through Q Excative HF Tune 2.4 and Xcalibur 3.0. MS spectra were acquired in the scan range of 375–1500 m/z with resolution of 60,000 at m/z 200, automatic gain control (AGC) target of 3∗10ˆ6, and a maximum injection time (IT) of 50 ms. The 12 most intense eluting peptides above intensity threshold 6∗10ˆ4, and charge states two or higher, were sequentially isolated for higher energy collision dissociation (HCD) fragmentation and MS2 acquisition to a normalized HCD collision energy of 32%, target AGC value of 1∗10ˆ5, resolution R = 60,000, and IT of 110 ms. The precursor isolation window was set to 1.6 m/z with an isolation offset of 0.3 and a dynamic exclusion of 30 s. Lock-mass (445.12003 m/z) internal calibration was used, and isotope exclusion was active. Raw data were analyzed by MaxQuant v1.5.5.1^38^ with "Variable Modifications" set for TMT 10-plex 126, 127N, 127C, 128N 128C, 129N, 129C, 130N, 130C, 131 to be at N-termini, as well as lysine for database searching and peptide identification.

### Quantification and statistical analysis

#### RNA sequencing quality control and transcript abundance estimation

FASTQ files were trimmed using Trimmomatic version 0.39^39^ to remove potential Illumina adapters and low quality bases with the following parameters: ILLUMINACLIP:truseq.fa:2:30:10 LEADING:3 TRAILING:3 SLIDINGWINDOW:4:15. FASTQ files were assessed using fastQC version 0.11.5^53^ prior to and following trimming. We used Salmon version 1.3.0^40^ to quantify the abundance at the transcript level with the fragment-level GC bias correction (option--gcBias) using the GENCODE Release 32 (GRCh38.p13) reference transcriptome and the GRCh38 reference genome, included as decoy.^54^ Transcript counts were collapsed to gene-level using R package tximport^41^ version 1.14.2 with default parameters (i.e., countsFromAbundances = FALSE) and the GENCODE Release 32 (GRCh38.p13) annotation. Henceforth, we use the notion of transcript in a gene-centric sense, i.e., as the entity defined by all transcript isoforms mapped to the same gene. mtDNA-encoded genes were removed from the analysis. Genes were further filtered out if unusually highly expressed (i.e., if they accounted for more than 1% of a sample’s library size in more than 50% of all the samples). We calculated log2 transformed counts per million (CPM) for the pre-filtered set of genes. Low-expressed genes (log2-CPM <0.1, in at least 80% of the samples) were also filtered out. The pre-filtered transcriptomic dataset resulted in a total of N = 29,959 genes.

#### Proteomics normalization and filtering

Aggregated protein intensities from maxQuant were further processed in a downstream analysis using R. First, proteins labelled as "Reverse", "Potential.contaminant" and "Only.identified.by.site" were removed from the analysis. In addition, proteins were removed if they exhibited at least one zero intensity in a sample. In order to filter out highly expressed proteins, we selected the top four highest expressed proteins in each sample (which ranged from 3% to 5% of the total expression of a sample). The union set of these (a total of 19 proteins) was then removed from all samples. We considered three possible normalization approaches for protein quantification, i) raw protein intensities, ii) quantile normalization, and iii) batch effect correction followed by root mean square scaling. To assess each of these strategies we explored the association of the first two components of the principal component analysis (PCA) of the protein expression matrix with the TMT batch. Raw protein intensities (i) showed a clear clustering of samples which was associated with the batches of the TMT experiment, which was further amplified by quantile normalization (ii). This effect was no longer noticeable when we applied batch correction as suggested in^55^ (iii), where we divided protein intensities by the correction factor based on the reference channels in the respective batches, followed by root mean square scaling (Figure S1). Additionally, we leveraged the transcriptomic samples to gain insight into the biological validity of the three alternative normalization options by studying the transcriptome-proteome correlation in the neurologically healthy groups (HA and YG; log2 transformed values for proteins, and log2 transcript CPMs). The transcriptome-proteome correlation was significantly higher in the batch-corrected strategy both across samples and across genes (Figure S2). Based on these observations we chose to apply the batch correction and subsequent root mean square scaling (iii). The pre-filtered proteomic dataset was composed of a total of N = 2,953 proteins. Preprocessing of proteomics data (filtering and normalization) was performed on a dataset that included additional samples not analyzed in this work (marked as “Other” in Figure S1). To perform batch correction, the inclusion of these samples was necessary. Downstream analyses (i.e., integration with transcriptomic data) were performed on the samples described (YG, HA, PD).

#### Covariance between omic layers

We used sparse partial least square (sPLS) as implemented in the mixOmics R package version 6.10.9^42^^,^^43^ to find the linear combinations of variables (transcripts and proteins) that maximize covariance between the transcriptomic and the proteomic layers. sPLS was performed on the pre-filtered transcriptomic (X) and proteomic (Y) datasets using the "canonical" mode and the parameters keepX = 50 and keepY = 50 for feature selection which is performed by the sPLS function.

#### Correlation between transcriptome and proteome

To increase the signal-to-noise ratio, genes were removed if they satisfied at least one of the following criteria: i) not present in the pre-filtered transcriptome, ii) not present in the pre-filtered proteome, iii) low median transcript expression (below 10% quantile), iv) low transcript variance (below 15% quantile). The removal of flagged genes resulted in an analysis-ready RNA-protein dataset of N = 2,104 genes. Gene-wise transcript-protein Pearson correlations were calculated across samples (resulting in one correlation coefficient per gene) independently for each group (HA, PD, YG) using log2 transformed CPMs for transcript abundance and log2 transformed batch-corrected and root mean square scaled protein intensities.

#### Gene scoring

For pathway enrichment analysis, genes were ranked according to the magnitude of change in correlation (δr) between the groups being compared. For example, when comparing YG to HA or HA to PD, each gene would be scored by δr = r_HA_–r_YG._, or δr = r_PD_ – r_HA_, respectively (Figure 2A). For each group comparison (YG->HA, YG->PD, HA->PD), we classified genes according to their change in transcript-protein correlation: a) *decoupling*: genes that show a positive transcript-protein correlation in the reference group and loose this correlation (r∼0) in the other group; b) *increased inverse correlation*: genes whichshow a correlation ≥0 in the reference group and a negative correlation in the other group; or c) *increased positive correlation*: genes with a correlation ≥0 in the reference group and an increased positive correlation in the other group (Figure 2B). To this end, gene-specific scores were calculated as follows:

$$\forall R_{ref}>\;0$$

$$S_{a}^{i}=-\left|R_{ageing}\right|+R_{ref}$$

$$S_{b}^{i}=-R_{ageing}+t\left(R_{ref}\right)$$

$$S_{c}^{i}=R_{ageing}–\;t\left(R_{ref}\right)\text{,}$$

with t(x)=(x+1)/2,

*where* i∈{1,2,3}

specifies the comparison being made

$$R_{ref}=\left\{ \begin{array}{cc} R_{YG}\text{,} & fori\in 1,2\\ R_{HA,} & fori=3 \end{array}\right.R_{ageing}=\left\{ \begin{array}{cc} R_{HA}\text{,} & fori=1\\ R_{PD}\text{,} & fori\in 2,3 \end{array}\right.$$

and *a*, *b*, and *c* specify the functional scenario (*decoupling*, *increased inverse correlation* and *increased positive correlation*, respectively). This resulted in 9 different gene scorings (Figure 2C). Heatmaps to visualize scoring distributions in Figure 2C were created with the R package ComplexHeatmap.^44^

#### Pathway enrichment analysis

The above gene scorings were used to test for functional enrichment. For this we used the function *multilevel_fgsea* from the R package fgsea, version 1.21.^45^ Specific parameters are documented in the code for the analysis (see data access). For each scoretype we ran the function on two genesets: i) a simplified list of genesets from the Gene Ontology (GO) database^56^ and ii) a list of genesets from KEGG, accessed through MSigDB.^57^ Both lists are available as “.gmt” files in the code repository. To generate a simplified, non-redundant GO list, pathways from the complete GO databases (CC, BP and MF) were clustered iteratively based on their similarity (Cohen’s kappa, κ) until no κ > 0.4.

We performed permutation analyses to evaluate the effect of group size on the pathway enrichment result. To this end, we generated 3 groups of sizes k = 4, k = 9, and k = 15 by randomly sampling k individuals irrespective of their label. The sizes of these groups were chosen to be equal to the sizes of the YG, HA and PD groups, respectively. This procedure was iterated N = 5,000 times. On each of the 5,000 permutation replicates we performed the same downstream analyses as with our original data: 1) we calculated correlation coefficients r across samples within each of the 3 groups; 2) we calculated gene scores to rank genes by their difference in r between groups according to our original analysis (Figure 2A). To evaluate the significance of our original results, we calculated the false discovery rate (FDR) as the fraction of permutation replicates that showed a more extreme enrichment score than our observation. We then defined a pathway as significant if FDR <0.1.

#### Protein interaction networks

Protein-protein interaction networks were generated using the R package coexnet version 1.8.0^46^ which retrieves information on protein co-expression and experimentally evidenced interaction from STRING.^47^ Vertices were clustered using the R package igraph version 1.2.5^48^ and its implemented edge-betweenness cluster algorithm.

## Data Availability

• The datasets supporting the conclusions of this article are included within the article and its supplementary files. Raw data and metadata have been deposited at Figshare and are publicly available as of the date of publication (Figshare: https://doi.org/10.6084/m9.figshare.21617886.v1). • The source code including description and all data for the analyses are available on GitHub: https://github.com/fifdick/alt_rna_prot_coupling_PD. All original code has been deposited at Zenodo and is publicly available as of the date of publication (Zenedo: https://doi.org/10.5281/zenodo.7357345). • Any additional information required to reanalyze the data reported in this paper is available from the lead contact upon request.

## Supplemental information

Document S1. Figures S1–S4 and Table S3

Table S1. Cohort demographic and experimental information, related to STAR Methods - Experimental model and subject details

Table S2. Correlation coefficients, related to Figure 2
