# Supplementary material for: Altered transcriptome-proteome coupling indicates aberrant proteostasis in Parkinson’s disease
Source: iScience. 2023 Jan 4;26(2):105925. doi: 10.1016/j.isci.2023.105925 (PMC9874017; doi:10.1016/j.isci.2023.105925)
Supplement: Document S1. Figures S1–S4 and Table S3 [file mmc1.pdf]

iScience, Volume *26*

Supplemental information

Altered transcriptome-proteome coupling indicates aberrant proteostasis in Parkinson's disease

Fiona Dick, Ole-Bjørn Tysnes, Guido W. Alves, Gonzalo S. Nido, and Charalampos Tzoulis

Supplemental figures

Fig S1 Principal component analysis visualized for all TMT samples. Related to STAR METHODS – Proteomics normalization and filtering.

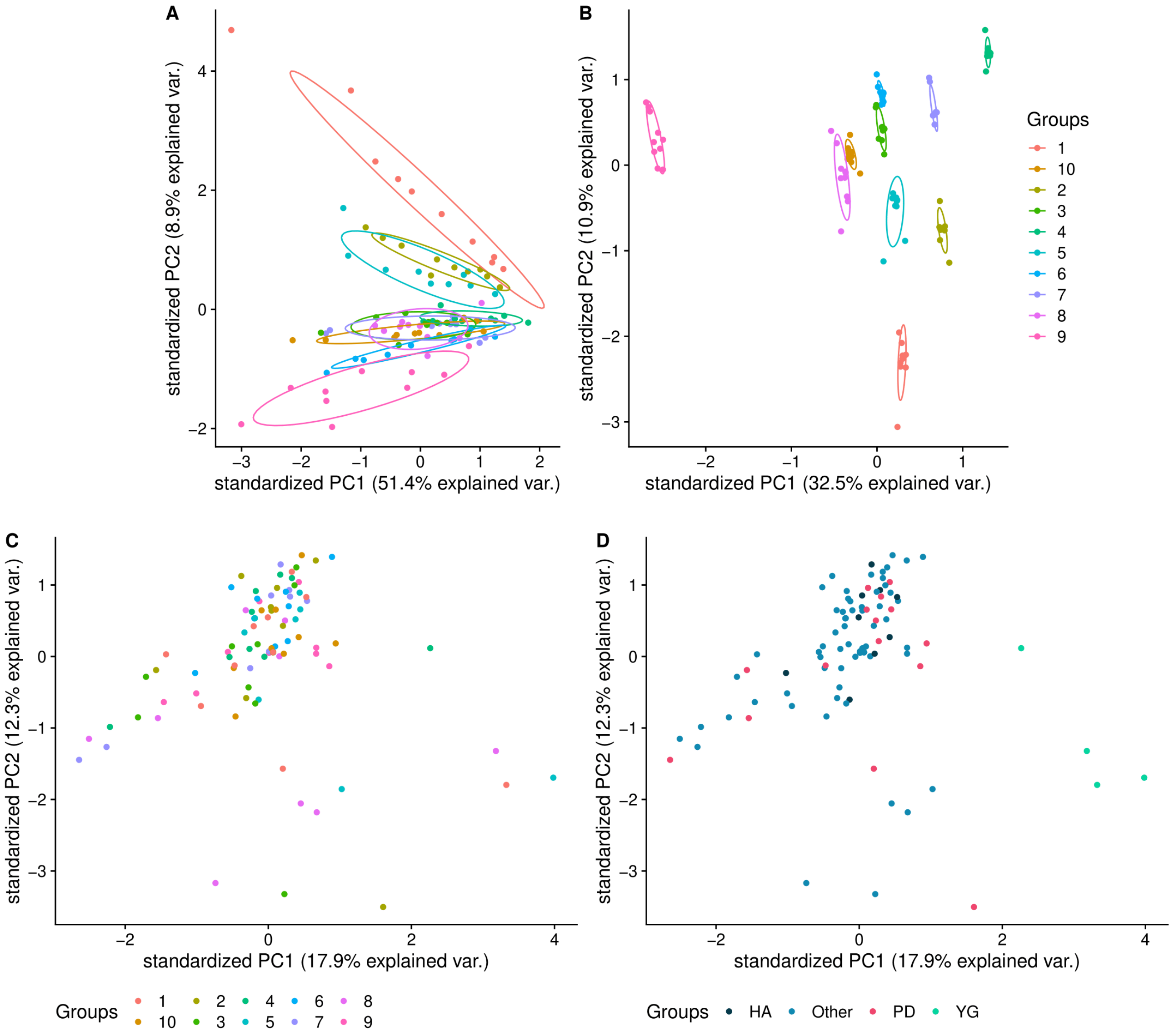

Fig S1 Principal component analysis visualized for all TMT samples. Related to STAR METHODS – Proteomics normalization and filtering.

Data points represent samples spanned by the first (x-axis) and second (y-axis) component of principal component analysis on raw protein intensities (A), quantile normalized protein intensities (B) and scaled batch corrected protein intensities (C and D). Coloring indicates the TMT batch of the sample for A, B and C and the sample's condition for D.

Fig S2 Batch correction of proteomics data improves correlation with RNA. Related to STAR METHODS – Proteomics normalization and filtering

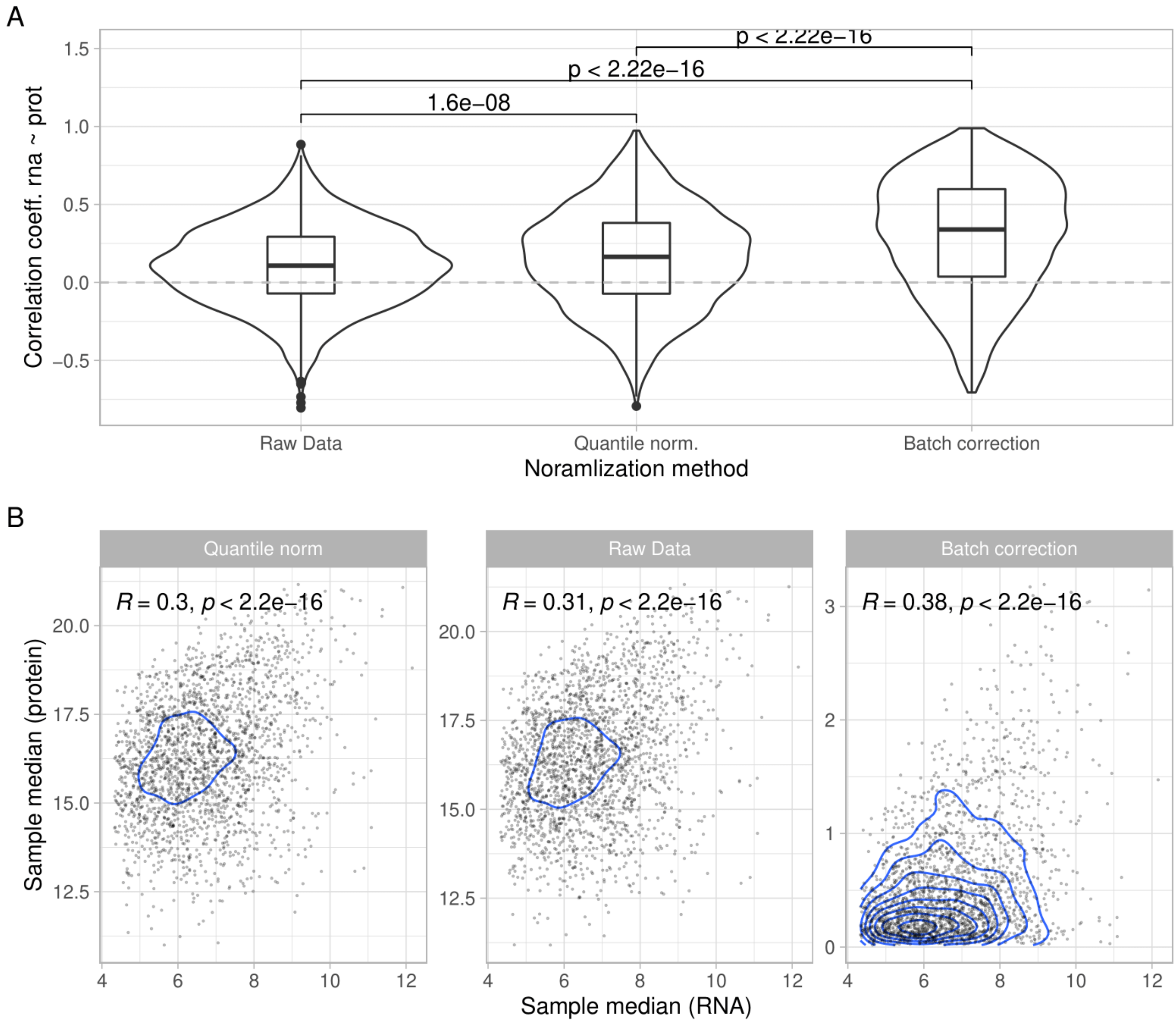

Fig S2 Batch correction of proteomics data improves correlation with RNA. Related to STAR METHODS – Proteomics normalization and filtering

A: Distribution of gene-wise correlation coefficients (RNA ~ protein) (y-axis) are displayed for the three normalization approaches of protein intensities (x-axis). B: Comparison of correlation between sample-median RNA expression (x-axis) and sample-median protein expression (y-axis) for the three different protein intensity normalization approaches (facets).

Fig S3 Distributions of RNA-protein correlations in a comparison between groups. Related to Figure 4

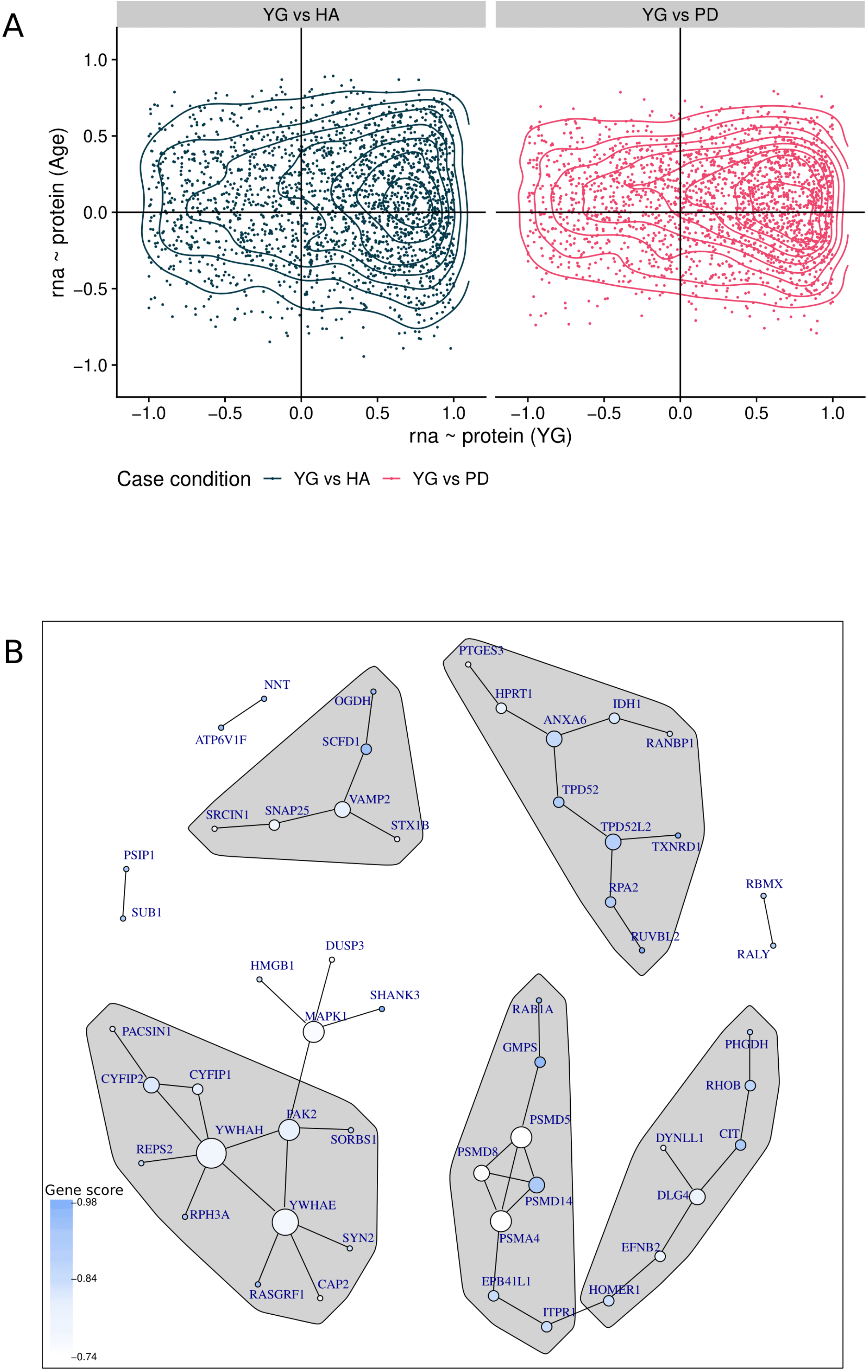
A
YG vs HA
YG vs PD
1.0
0.5
0.0
−0.5
−1.0
rna ~ protein (Age)
−1.0
−0.5
0.0
0.5
1.0
rna ~ protein (YG)
Case condition
YG vs HA
YG vs PD
B
NNT
ATP6V1F
OGDH
SCFD1
VAMP2
SNAP25
SRCIN1
STX1B
PTGES3
HPRT1
ANXA6
IDH1
RANBP1
TPD52
TPD52L2
TXNRD1
RPA2
RUVBL2
RBMX
RALY
PSIP1
SUB1
DUSP3
HMGB1
SHANK3
MAPK1
PACSIN1
CYFIP2
CYFIP1
YWHAH
PAK2
SORBS1
REPS2
RPH3A
YWHAE
SYN2
RASGRF1
CAP2
RAB1A
GMPS
PSMD5
PSMD8
PSMD14
PSMA4
EPB41L1
ITPR1
PHGDH
RHOB
DYNLL1
CIT
DLG4
EFNB2
HOMER1
Gene score
−0.98
−0.84
−0.74

Fig S3 Distributions of RNA-protein correlations in a comparison between groups. Related to Figure 4

A: Two-dimensional density plot displaying both distribution and relationship between the reference YG (x-axis) and the ageing groups (y-axis): YG vs HA (dark blue, first panel), and YG vs PD (pink, second panel). B: Protein-protein interaction (PPI) network for genes in the 0.90 quantile of gene-scores (blue), ranking genes by decoupling in PD. Only genes that have at least one edge are displayed. Vertex communities were identified using edge betweenness (R packageigraph). Only communities with more than 5 members are displayed. PPI is based on coexpression, experimental evidence of interaction and neighbourhood characteristics

Fig S4 Distribution of correlation coefficients influenced by group size – Related to Figure 4

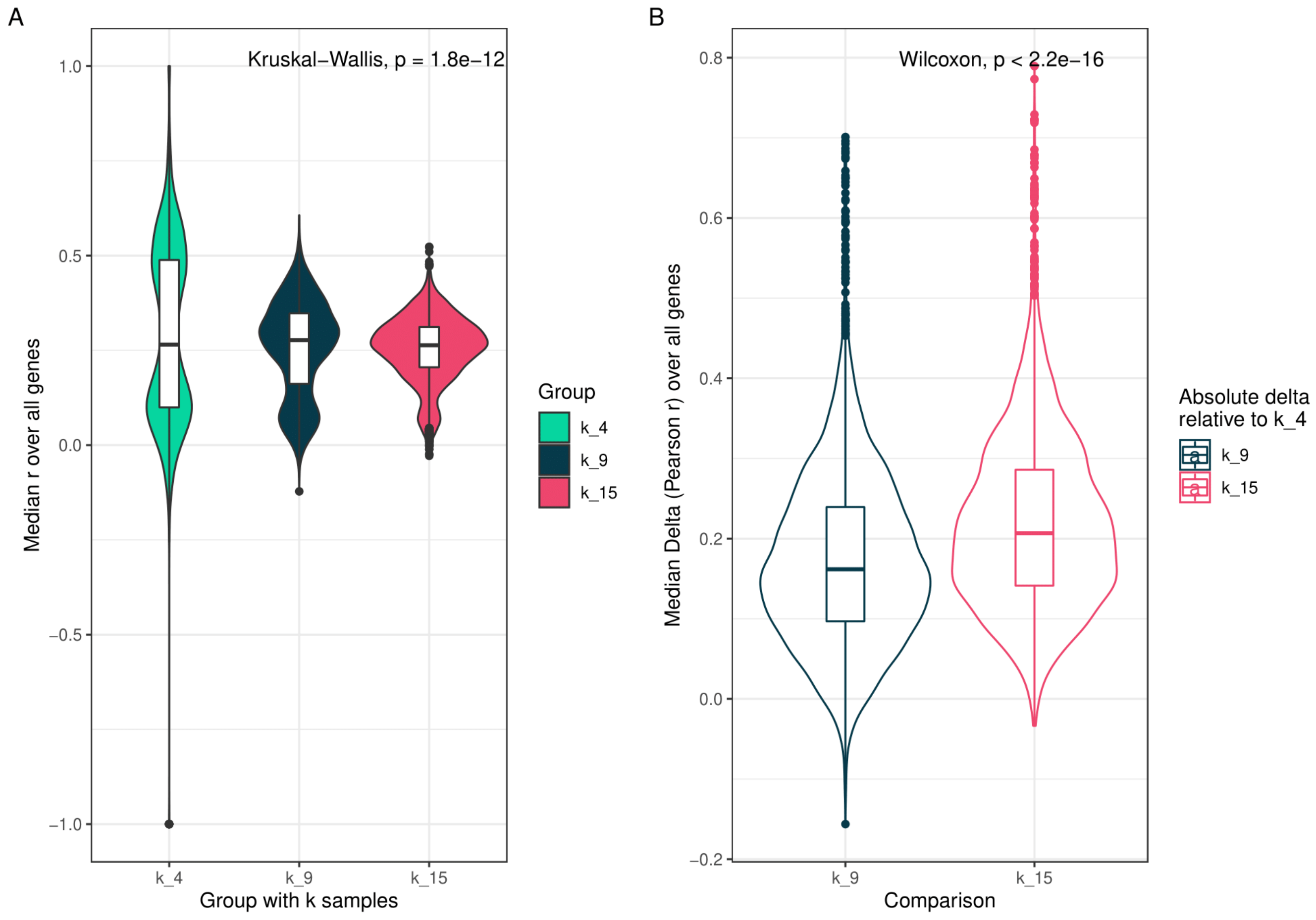

Fig S4 Distribution of correlation coefficients influenced by group size – Related to Figure 4

A: Group-wise distribution of mean mRNA-protein correlation (y-axis) over all genes of each permutation replicate. In each permutation, groups (x-axis) are formed by sampling k samples with replacement, irrespective of their true label. Color coding indicates group membership, k indicates group size: k_4 (turquoise), k_9 (dark blue), k_15 (pink). B: Distribution of the mean deltas (differences in absolute r) over all genes of each permutation replicate. Delta is calculated for k_9 (dark blue) and k_15 (pink) relative to k_4.

Supplemental table

Table S3 Pathway enrichment on genes ranked by RNA-protein correlation coefficient – Related to Table 1

Ranked by	Pathway	Adj. P-value	Enrichment score	Ontology	Permutation p-value
r_{PD}	KEGG_PARKINSONS_DISEASE	0.0181	0.3981	KEGG	0.0515
r_{PD}	KEGG_ALZHEIMERS_DISEASE	0.0184	0.3592	KEGG	0.0640
r_{PD}	KEGG_OXIDATIVE_PHOSPHORYLATION	0.0184	0.3746	KEGG	0.0575
r_{PD}	KEGG_HUNTINGTONS_DISEASE	0.0214	0.3490	KEGG	0.0580
r_{PD}	KEGG_PROXIMAL_TUBULE_BICARBONATE_RECLAMATION	0.0495	0.5720	KEGG	0.0040
r_{PD}	cellular respiration	0.0102	0.3786	GO	0.0560
r_{PD}	aerobic respiration	0.0141	0.4502	GO	0.0370
$-r_{PD}$	KEGG_PROTEASOME	0.0004	0.5763	KEGG	0.0190
$-r_{PD}$	antigen processing and presentation of peptide antigen via MHC class I	0.0009	0.5462	GO	0.0390
$-r_{PD}$	NIK/NF-kappaB signaling	0.0010	0.5426	GO	0.0320
$-r_{PD}$	anaphase-promoting complex-dependent catabolic process	0.0010	0.5574	GO	0.0235
$-r_{PD}$	cytoplasmic translation	0.0010	0.5062	GO	0.0815
$-r_{PD}$	innate immune response-activating signal transduction	0.0010	0.4602	GO	0.0165
$-r_{PD}$	tumor necrosis factor-mediated signaling pathway	0.0010	0.5263	GO	0.0275
$-r_{PD}$	negative regulation of cell cycle G2/M phase transition	0.0013	0.5180	GO	0.0510
$-r_{PD}$	interleukin-1-mediated signaling pathway	0.0015	0.5020	GO	0.0305
$-r_{PD}$	innate immune response activating cell surface	0.0021	0.4865	GO	0.0345

	receptor signaling pathway				
**$-r_{PD}$**	regulation of RNA stability	0.0021	0.4550	GO	0.0375
**$-r_{PD}$**	polysome	0.0024	0.4887	GO	0.1025
**$- r_{PD}$**	positive regulation of canonical Wnt signaling pathway	0.0024	0.4800	GO	0.0415
**$- r_{PD}$**	translational initiation	0.0024	0.3799	GO	0.1405
**$- r_{PD}$**	proteasomal ubiquitin-independent protein catabolic process	0.0026	0.6719	GO	0.0070
**$- r_{PD}$**	regulation of hematopoietic progenitor cell differentiation	0.0026	0.5103	GO	0.0475
**$- r_{PD}$**	SCF-dependent proteasomal ubiquitin-dependent protein catabolic process	0.0029	0.4918	GO	0.0540
**$- r_{PD}$**	antigen processing and presentation of peptide antigen	0.0029	0.4048	GO	0.0805
**$- r_{PD}$**	unfolded protein binding	0.0061	0.4227	GO	0.0860
**$- r_{PD}$**	viral gene expression	0.0061	0.3840	GO	0.1385
**$- r_{PD}$**	regulation of DNA-templated transcription in response to stress	0.0063	0.4575	GO	0.0745
**$- r_{PD}$**	peptidase complex	0.0080	0.4464	GO	0.0395
**$- r_{PD}$**	response to interleukin-1	0.0083	0.4233	GO	0.0410
**$- r_{PD}$**	ribosomal subunit	0.0095	0.3824	GO	0.1365
**$- r_{PD}$**	regulation of cellular amine metabolic process	0.0106	0.4428	GO	0.0570
**$- r_{PD}$**	protein localization to endoplasmic reticulum	0.0147	0.3595	GO	0.1450
**$- r_{PD}$**	negative regulation of canonical Wnt signaling pathway	0.0147	0.4320	GO	0.0685
**$- r_{PD}$**	positive regulation of ATPase activity	0.0147	0.4915	GO	0.0745
**$- r_{PD}$**	ATPase activator activity	0.0214	0.6031	GO	0.1245
**$- r_{PD}$**	translation initiation factor activity	0.0214	0.5132	GO	0.0435

**- r_{PD}**	nuclear-transcribed mRNA catabolic process, nonsense-mediated decay	0.0220	0.3603	GO	0.1505
**- r_{PD}**	protein folding	0.0220	0.3513	GO	0.0960
**- r_{PD}**	nuclear chromosome, telomeric region	0.0274	0.5787	GO	0.0405
**- r_{PD}**	cytoplasmic translational initiation	0.0276	0.5917	GO	0.0120
**- r_{PD}**	regulation of morphogenesis of an epithelium	0.0300	0.4071	GO	0.1600
**- r_{PD}**	non-canonical Wnt signaling pathway	0.0321	0.4053	GO	0.1735
**- r_{PD}**	ribosome binding	0.0376	0.5535	GO	0.1585
**- r_{PD}**	cell redox homeostasis	0.0389	0.4974	GO	0.0205
**- r_{PD}**	chaperone binding	0.0389	0.4092	GO	0.4175
**- r_{PD}**	polysomal ribosome	0.0459	0.4788	GO	0.1385
**- r_{HA}**	KEGG_RIBOSOME	0.0000	0.4795	KEGG	0.0810
**- r_{HA}**	nuclear-transcribed mRNA catabolic process, nonsense-mediated decay	0.0002	0.4577	GO	0.0865
**- r_{HA}**	ribosomal subunit	0.0002	0.4668	GO	0.0930
**- r_{HA}**	protein localization to endoplasmic reticulum	0.0002	0.4408	GO	0.0930
**- r_{HA}**	translational initiation	0.0004	0.4207	GO	0.1285
**- r_{HA}**	viral gene expression	0.0012	0.4276	GO	0.1225
**- r_{HA}**	synaptic vesicle	0.0120	0.3886	GO	0.3195
**- r_{HA}**	cytoplasmic translation	0.0438	0.4520	GO	0.1530

Table S3 Pathway enrichment on genes ranked by RNA-protein correlation coefficient – Related to Table 1

Pathways are sorted within each ranking (“Ranked by”) and ontology (GO or KEGG) by their enrichment score. Permutation p-value represents the fraction of permutations (from randomly sampled data) for which the enrichment score was greater than the enrichment score of the observation.
